# Selective Sensing of Tyrosine Phosphorylation in Peptides Using Terbium(III) Complexes

**DOI:** 10.1155/2016/3216523

**Published:** 2016-06-08

**Authors:** Jun Sumaoka, Hiroki Akiba, Makoto Komiyama

**Affiliations:** ^1^Department of Applied Chemistry, School of Engineering, Tokyo University of Technology, 1404-1 Katakuramachi, Hachioji, Tokyo 192-0982, Japan; ^2^Research Center for Advanced Science and Technology, The University of Tokyo, 4-6-1 Komaba, Meguro-ku, Tokyo 153-8904, Japan; ^3^Life Science Center of Tsukuba Advanced Research Alliance, University of Tsukuba, 1-1-1 Ten-noudai, Tsukuba, Ibaraki 305-8577, Japan; ^4^Department of Bioengineering, School of Engineering, The University of Tokyo, 7-3-1 Hongo, Bunkyo-ku, Tokyo 113-8656, Japan

## Abstract

Phosphorylation of tyrosine residues in proteins, as well as their dephosphorylation, is closely related to various diseases. However, this phosphorylation is usually accompanied by more abundant phosphorylation of serine and threonine residues in the proteins and covers only 0.05% of the total phosphorylation. Accordingly, highly selective detection of phosphorylated tyrosine in proteins is an urgent subject. In this review, recent developments in this field are described. Monomeric and binuclear Tb^III^ complexes, which emit notable luminescence only in the presence of phosphotyrosine (pTyr), have been developed. There, the benzene ring of pTyr functions as an antenna and transfers its photoexcitation energy to the Tb^III^ ion as the emission center. Even in the coexistence of phosphoserine (pSer) and phosphothreonine (pThr), pTyr can be efficintly detected with high selectivity. Simply by adding these Tb^III^ complexes to the solutions, phosphorylation of tyrosine in peptides by protein tyrosine kinases and dephosphorylation by protein tyrosine phosphatases can be successfully visualized in a real-time fashion. Furthermore, the activities of various inhibitors on these enzymes are quantitatively evaluated, indicating a strong potential of the method for efficient screening of eminent inhibitors from a number of candidates.

## 1. Introduction

In nature, enzymatic phosphorylation and dephosphorylation of proteins control many biological events. Cellular pathways regulated by these enzymatic modifications of proteins are so versatile. In the course of signal transduction in cells, for example, Ser, Thr, and Tyr, residues in proteins are reversibly phosphorylated and dephosphorylated, resulting in desired modulation of the activity of relevant enzymes [1, 2]. In terms of the importance of these enzymatic reactions, a number of elegant chemical sensors to detect them in proteins have been already reported. In most of these sensors, phosphate residue(s) of phosphoserine (pSer), phosphothreonine (pThr), and phosphotyrosine (pTyr) in proteins is selectively bound as the recognition target so that these three types of phosphorylations are detected at similar sensitivity without significant discrimination [3–11]. Valuable information on the roles of protein phosphorylations in biological systems has been obtained. The molecular designs of these sensors and their practical applications have been the subjects of many excellent reviews [12–21].

**Figure 1:**
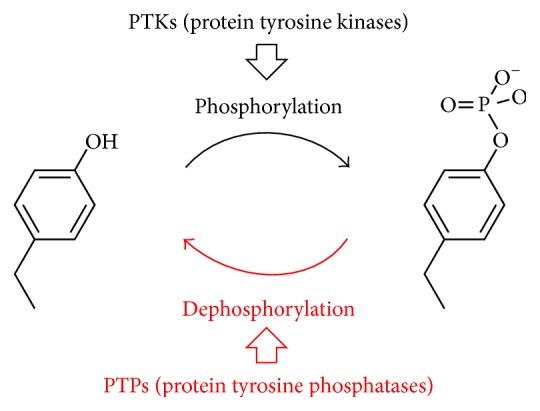
Phosphorylation of tyrosine residue by protein tyrosine kinases (PTKs) and its dephosphorylation by protein tyrosine phosphatases (PTPs) for the regulation of biological functions of proteins.

In contrast with these overall detections of phosphorylations of Ser, Thr, and Tyr in proteins, this review focuses on selective detection of phosphorylation of Tyr alone (Figure 1). This Tyr phosphorylation by protein tyrosine kinases (PTKs) and protein tyrosine phosphatases (PTPs) accounts for only 0.05% of the total phosphorylation in cells (the majority of phosphorylation occurs on Ser or Thr) but takes a crucial role in the regulation of highly important biological functions (differentiation, adhesion, cycle control, endocytosis, and many others) [22, 23]. In epidermal growth factor receptor (EGFR), its autophosphorylation of a Tyr residue triggers signal-cascade in cells [24, 25]. In the downstream, there work several Src family kinases, which are also controlled by their Tyr phosphorylations and in turn phosphorylate Tyr residues in other proteins [26–28]. If Tyr phosphorylation is excessive or insufficient, serious problems are induced to the living. Therefore, PTKs and PTPs are regarded as main targets in drug discovery [29–34]. For many years, a number of laboratories developed elegant optical sensors to evaluate the activities of these enzymes. In some of them, substrate peptide was conjugated (or fused) to a probe molecule (e.g., Tb(III) complexes [35–40], Mg(II) complexes [41–47], Ca(II) complex [48], Zn(II) complex [49], Cd(II) complex [50], peptide derivatives [51, 52], and others [53, 54]). The other sensors involve noncovalent interactions between a substrate and a probe (e.g., Tb(III) ion [55–62], Eu(III) complex [63, 64], platinum(II) complex [65], and Tb(III) complexes [66–69]).

Among all the probes investigated, lanthanide ions and their complexes have been widely and successfully employed due to their unique light-emitting properties [70–77]. The photoluminescence from these ions has unusually long life-time (in the order of micro- to milliseconds), and thus the background signal can be minimized with the use of time-resolved spectroscopy. Alternatively, the kinase reactions were followed by the disappearance of ATP (source of the phosphate group for pTyr) [78, 79], whereas the phosphatase functions were monitored by the production of phosphoric acid [80]. However, these analytical methods are often complicated by the perturbation signals from other phosphate-containing solutes, ATP-dependent reactions, and/or phosphate-producing processes in the specimens. In addition to these chemical sensors, antibodies specific to pTyr are widely being used at present for practical applications, but their usage has been hampered by high costs, rather poor stability, and other factors. Accordingly, chemical probes that directly visualize PTK/PTP activity and produce unbiased signals are required for further developments of the field.

This paper reviews recent developments in optical methods to selectively detect pTyr in proteins. The primary concerns are high sensitivity of pTyr detection and its sufficient specificity (with respect to pSer and pThr, which exist much more abundantly in biological systems). As emission probes, lanthanide ions (especially Tb^III^ ion) and their complexes are used. By combining unique properties of the emission from these metal ions with so-called “antenna effect,” the background signals are minimized, and only the signal from pTyr is selectively monitored [67]. The detection activity on pTyr is further promoted by forming binuclear Tb^III^ complexes [68]. With the use of these chemical sensors, phosphorylation of peptides by PTKs and their dephosphorylation by PTPs are followed in a real-time fashion [38, 68, 69]. Applications of the methods to screening of efficient inhibitors on PTKs and PTPs are also presented.

## 2. Principle of Selective Detection of pTyr by Tb(III) Complexes

The emission from lanthanide ions is intrinsically weak, since the corresponding f-f transitions are Laporte-forbidden. However, this luminescence is enormously strengthened, when a chromophore (“antenna”) is placed near the lanthanide ions and transfers its photoexcitation energy to these emission centers [70–77, 81–91]. By combining lanthanide complexes with antenna molecules, elegant systems to detect various anionic guests have been already prepared. Sophisticated examples include the analysis of carboxylic acid derivatives [85–100], halide ions [95–98, 101–108], nitrate ions [96, 97, 101–105, 109], and hydrogen sulfate ion [110]. Furthermore, phosphate ion [95–99, 108–116], pyrophosphate [113–115], ATP [113–115, 117–121], and other molecules containing phosphate [120–122] were also detected by using lanthanide complexes. Upon the binding of the phosphate group(s) to the complexes, the chemical environments around the lanthanide(III) ions were altered, inducing a change in the luminescent property of the ions. With this strategy, however, highly selective detection of pTyr is rather difficult, since coexisting phosphate groups in solutions (e.g., pSer, pThr, ATP, and DNA) could show similar effects [123–125]. These factors are more critical when a Tb^III^ ion (without any ligand) is used; note that nucleotides and nucleic acids are also eminent antenna (*vide infra*) [55, 56].

**Figure 2:**
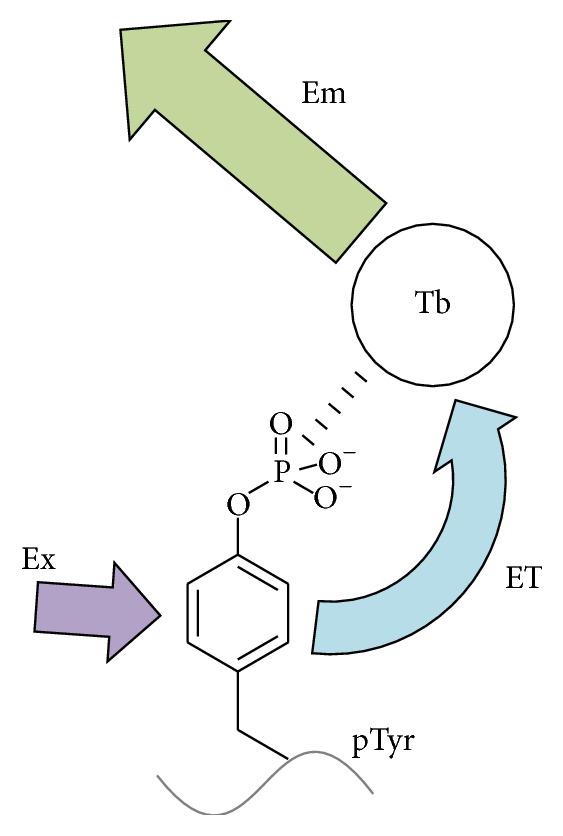
Mechanism of selective detection of enzymatic phosphorylation of Tyr by Tb^III^ complex. The photoexcited energy (Ex) absorbed by the benzene ring (antenna) is transferred to the Tb^III^ (ET), resulting in enormous promotion of the intensity of luminescence emitted from this metal ion (Em). Accordingly, the emission is evident only for pTyr which fulfills both of the two requirements for the mechanism (notable antenna effect and sufficient binding activity towards the Tb^III^).

One successful solution to these problems (improvement of the selectivity of pTyr detection with respect to (i) nonphosphorylated Tyr, (ii) pSer and pThr, and (iii) other coexisting phosphate-containing biomolecules) is presented in Figure 2. This strategy, developed in our laboratory [67], is based on the fact that both the benzene ring and the phosphate group are definitely required for the efficient photoluminescence. First, the benzene ring of pTyr in the target peptides is used as an antenna to enhance the emission from the Tb^III^ center. The irradiated light (Ex) is first absorbed by this benzene ring, and the excitation energy is then transferred to Tb^III^ (ET). Finally, the metal center emits luminescence from its photoexcited state (Em). On the other hand, the phosphate is essential to bind to Tb^III^ and places the benzene ring near the metal ion as the emission center. Among the coexisting solutes (Tyr, Ser, Thr, and their phosphorylated products), only pTyr possesses both notable antenna effect (benzene ring) and sufficient binding activity towards the Tb^III^ complex (phosphate group). Thus, the selectivities (i) and (ii) are fulfilled. Furthermore, the selectivity (iii) to other coexisting phosphate-containing molecules is accomplished by using a bulky ligand which suppresses the access of these molecules to Tb^III^. Furthermore, nonspecific background signals can be removed by using time-resolved spectroscopy and analyzing only long life-time components of the luminescence emitted from Tb^III^.

## 3. Selective Detection of Enzymatic Phosphorylation of Tyr by Tb^**III**^ Complex-Based Sensors

Based on the strategy depicted in Figure 2, enzymatic phosphorylations and dephosphorylations of Tyr were monitored by using Tb^III^ complexes. In the first part of Section 3.1, a monomeric Tb^III^ complex was prepared primarily to show the validity of the working hypothesis. In Section 3.2, the sensitivity of pTyr detection has been greatly enhanced by forming binuclear Tb^III^ complexes. As a result, useful tools to monitor enzymatic phosphorylation of Tyr (and its dephosphorylation) have been obtained and used for practical applications in the following sections. Among lanthanide ions, Tb^III^ has been most widely employed, together with Eu(III), for biological applications, emitting the most intensive line at around 545 nm.

### 3.1. Monomeric Tb^III^ Complex Showing Sufficient Selectivity for pTyr Detection [67]

**Figure 3:**
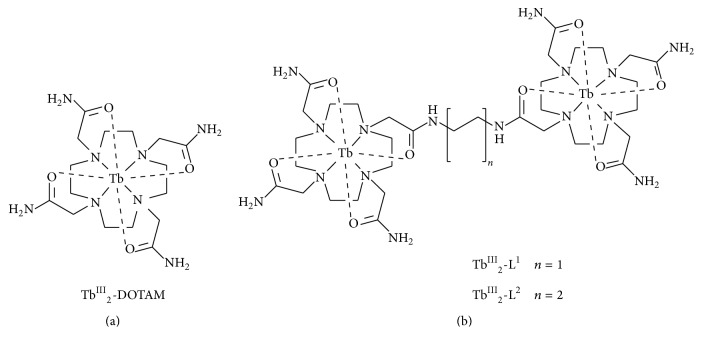
Structures of mononuclear DOTAM-Tb^III^ complex (a) and binuclear complexes Tb^III^_2_-L^1^ and Tb^III^_2_-L^2^ (b) used for selective detection of pTyr.

The sample solutions used for the sensing of pTyr always contain many other biological molecules, and some of them show notable antenna effects to induce the emission from lanthanide ions. Among them, nucleobases and nucleic acids especially deserve attention. For example, guanosine 5′-monophosphate (GMP) enormously enhances the luminescence through the binding to Tb^III^ ion by multicoordination of both the phosphate and the guanine (N7 and O6). Thus, Tb^III^ ion itself is not directly applicable to the sensing. In order to suppress the emission due to these coexisting molecules and accomplish efficient sensing of Tyr phosphorylation, an appropriate ligand is necessary to prevent the access of these molecules to Tb^III^ ion. For this purpose, DOTAM (2,2′,2′′,2′′′-(1,4,7,10-tetraazacyclododecane-1,4,7,10-tetrayl)tetraacetamide) was used (Figure 3(a)). This well-known ligand for lanthanide ions has no aromatic ring to work as antenna [126–129], and its Tb^III^ complexes have +3 net charges which are favourable to bind negatively charged pTyr. According to the design, the bulkiness of DOTAM should sterically interfere with the interactions between bulky nucleobases (or nucleic acids) and Tb^III^. On the other hand, the effect of DOTAM on the binding of the phosphate in pTyr to the Tb^III^ is little (or in much smaller magnitude) because of its smaller size.

**Figure 4:**
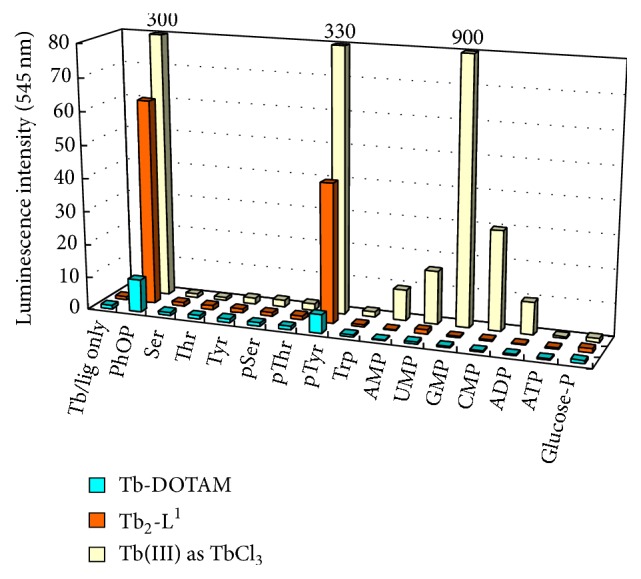
The luminescence intensity at 545 nm of Tb^III^-DOTAM (blue bars) and Tb^III^_2_-L^1^ (red bars) in the presence of various phosphorylated and nonphosphorylated amino acids, nucleoside derivatives, and PhOP (a model compound of pTyr). Conditions: [Tb^III^ complex] = [additive] = 100 *μ*M, pH 7.0 (10 mM HEPES buffer), *λ*_ex_ = 262.5 nm. For the purpose of comparison, the results using Tb^III^ ion without ligand are also presented (yellow bars). Note that nucleotides (UMP, GMP, CMP, and ADP) showed notable signals and thus selective detection of pTyr was unsuccessful.

Exactly as designed, pTyr notably increased the intensity of luminescence from Tb^III^-DOTAM complex (blue bars in Figure 4). Apparently, the phosphate residue of pTyr satisfactorily interacted with the complex despite the bulkiness of DOTAM, and the excitation energy of the benzene ring was efficiently transferred to the emission metal center. In contrast, GMP, as well as other nucleotides and nucleic acids, hardly promoted the luminescence from Tb^III^-DOTAM. Furthermore, nonphosphorylated Tyr, pSer, and pThr induced only marginal increase, as expected from the mechanism in Figure 2. The effect of either phenylalanine or tryptophan was negligible. Thus, Tb^III^-DOTAM complex is sufficiently effective in detecting pTyr selectively even in the coexistence of various analytes which otherwise produce undesirable noises of nonnegligible intensity. Using this complex, the tyrosine phosphorylation in a nonapeptide (^Ac-^Glu-Glu-Glu-Ile-Tyr-Glu-Glu-Phe-Asp^-CONH2^; P1 peptide [130]) was successfully monitored with a high signal-to-noise ratio. The mode of interaction between the metal center in Tb^III^-DOTAM and the phosphate group of pTyr was investigated by using phenyl phosphate (PhOP) as a model compound of pTyr (note that it also notably increased the luminescence from the DOTAM complex in Figure 4). In the presence or the absence of PhOP, the *q* value (the number of coordinated water molecules on Tb^III^) was determined by luminescence life-time measurements. Interestingly and importantly, *q* value was always around 1, whether or not PhOP was binding to the Tb^III^-DOTAM complex. In other words, one water molecule was originally coordinated to Tb^III^ in the Tb^III^-DOTAM complex, and this water molecule was never removed from Tb^III^ when the Tb^III^ complex interacted with PhOP. Thus, it has been concluded that the interaction between Tb^III^-DOTAM and PhOP (and thus pTyr also) is an ion-pairing rather than direct coordination of the phosphate to Tb^III^. Nevertheless, the benzene ring of pTyr is placed in a sufficient proximity of Tb^III^ and satisfactorily works as antenna.

### 3.2. Binuclear Tb^III^ Complexes for Promoted Detection Sensitivity on pTyr [68]

As shown in the previous section, mononuclear Tb^III^ complex has eminent selectivity for pTyr detection. However, the sensitivity is still rather limited, primarily because of its poor binding of phosphate group. In order to further increase the detection sensitivity of Tb^III^-DOTAM on pTyr, its binuclear complexes (Tb^III^_2_-L^1^ and Tb^III^_2_-L^2^) were developed (Figure 3(b)). In the ligands used, two DOTAM groups were connected by appropriate linkers of different length. These complexes, as well as Tb^III^-DOTAM, show intrinsically minimal luminescence in the absence of pTyr (no antenna moiety is available). Importantly, the luminescence from these binuclear Tb^III^ complexes was greatly enhanced when pTyr was added to the solution (red bars in Figure 4). Moreover, the pTyr-induced enhancements of luminescence from these binuclear complexes were far greater than pTyr-induced enhancement of luminescence from the mononuclear complex Tb^III^-DOTAM (compare the red bar with the blue bar in Figure 4). The origins of remarkable enhancements for the binuclear Tb^III^ complexes were investigated in detail using a model compound PhOP in place of pTyr. When [Tb^III^ complex] = [PhOP] = 100 *μ*M, the luminescence from Tb^III^_2_-L^1^ is stronger than that from Tb^III^-DOTAM by more than 10-fold. By analyzing the relationship between the luminescence intensity and the concentration of Tb^III^ complex in terms of Michaelis-Menten type equation, the dissociation constant of Tb^III^_2_-L^1^/PhOP complex was determined to be 29 *μ*M. This value was 110 times smaller than the corresponding value for Tb^III^-DOTAM. Thus, the binuclear Tb^III^ complexes have superior photoemission activity, mainly because they bind PhOP (and thus pTyr also) more efficiently. Apparently, the doubled positive charges of these binuclear complexes (+6) are responsible for the tighter interactions with the negatively charged phosphate of pTyr (note that the electrostatic interactions are primarily responsible for the binding;* vide ante*). Furthermore, the Tb^III^ center and the benzene ring of pTyr are in sufficient proximity for energy-transfer to occur smoothly. In addition to these enhancements in fluorescence intensity, the selectivity of pTyr detection of the binuclear Tb^III^ complexes, with respect to other cosolutes in solutions, is kept sufficiently high and comparable with that of the mononuclear Tb^III^-DOTAM. By using these binuclear Tb^III^ complexes, Tyr-phosphorylated nonapeptide (P1-pY) was clearly distinguished at pH 7 from nonphosphorylated nonapeptide (P1).

## 4. Real-Time Monitoring of Enzymatic Tyrosine Phosphorylation and Dephosphorylation [68, 69]

**Figure 5:**
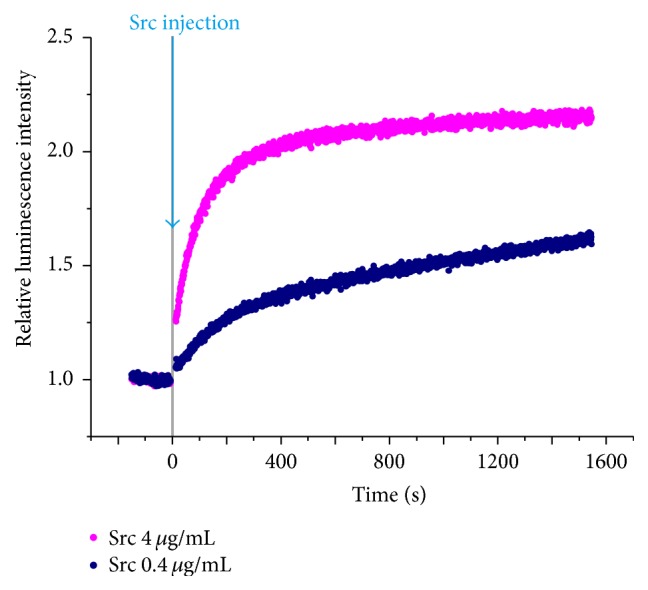
Time-dependent change of the luminescence intensity from Tb^III^_2_-L^1^ for the phosphorylation of P1 by protein tyrosine kinase Src. At time = 0, Src kinase was added to the solution containing other species and the reaction was started. [Src] = 4 (pink) and 0.4 *μ*g/mL (navy). [P1] = 5 *μ*M, [Tb^III^_2_-L^1^] = 100 *μ*M, [ATP] = 5 *μ*M, and [MnCl_2_] = 1 mM. The excitation at 262.5 nm and the emission at 545 nm. Reprinted with permission from [69]. Copyright 2014, Springer.

By using Tb^III^_2_-L^1^, the time-course of Tyr phosphorylation of peptides by PTKs can be straightforwardly monitored in real-time. For example, phosphorylation of the tyrosine residue in the center of a nonapeptide P1 by Src kinase was analyzed in Figure 5. To the solution containing Tb^III^_2_-L^1^ and P1, as well as ATP and MnCl_2_ (essential factors in this enzymatic reaction), Src tyrosine kinase was added and then the luminescence at 545 nm (^5^D_4_ → ^7^F_5_ transition) was measured. The luminescence intensity increased time-dependently, reflecting the Tyr phosphorylation. The magnitude of increase in luminescence intensity is exactly consistent with the difference in the concentration of P1. Without the substrate peptide, the luminescence was never enhanced. When Tyr-phosphorylated P1 was used as the substrate, the luminescence was strong from the beginning and not enhanced even after the addition of Src kinase. In order to confirm the validity of the method furthermore, the rate of this enzymatic phosphorylation was independently determined using TAMRA-labeled P1. There, the reaction was stopped at several reaction times and the products were analyzed by polyacrylamide gel-electrophoresis. The results of these two methods fairly agreed with each other, as expected.

**Figure 6:**
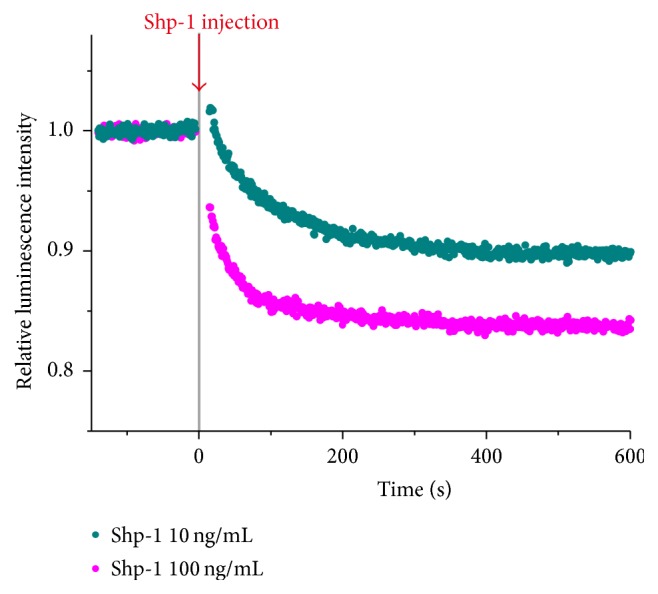
Time-dependent change of the luminescence intensity from Tb^III^_2_-L^1^ for the dephosphorylation of P1-pY by Shp-1 tyrosine phosphatase. [Shp-1] = 100 (pink) and 10 ng/mL (cyan). [P1-pY] = 10 *μ*M and [Tb^III^_2_-L^1^] = 100 *μ*M. The excitation at 262.5 nm and the emission at 545 nm. Reprinted with permission from [69]. Copyright 2014, Springer.

**Figure 7:**
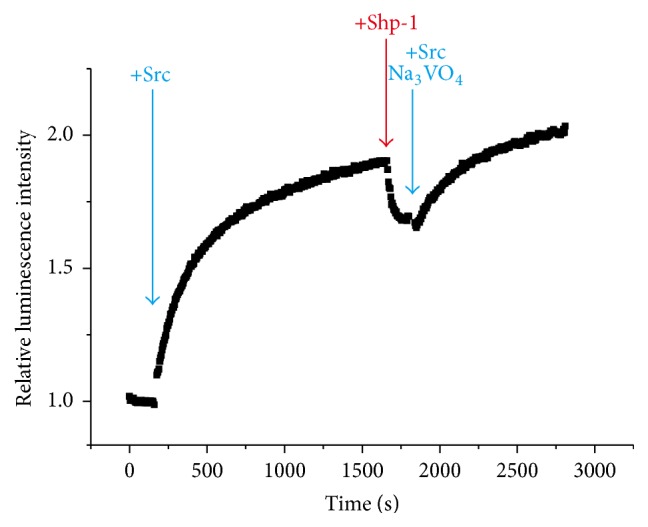
One-pot visualization by Tb^III^_2_-L^1^ of the sequential reactions of a PTK (Src kinase) and a PTP (Shp-1 phosphatase). To the reaction solution, Src kinase, Shp-1, and Na_3_VO_4_ (inhibitor of Shp-1) were added at the timings shown by the arrows. Reprinted with permission from [69]. Copyright 2014, Springer.

Similarly, the reverse reactions of the phosphorylations, dephosphorylations of a tyrosine-phosphorylated peptide by PTPs, were also visualized by Tb^III^_2_-L^1^ in real-time (Figure 6). Here, the peptide as kinase substrate was simply substituted with the corresponding phosphorylated peptide. When Shp-1 tyrosine phosphatase was added to the solution containing both P1-pY and Tb^III^_2_-L^1^, the luminescence intensity gradually decreased. The magnitude of luminescence change was exactly dependent on PTP concentration. The Tb^III^_2_-L^1^ binds relatively weakly to the pTyr residue and does not much disrupt the phosphatase reactions. Still more complicated sequential reactions of PTK and PTP were also monitored in one-pot fashion (Figure 7). When nonphosphorylated P1 was first phosphorylated by Src kinase, the luminescence intensity gradually increased. Then (e.g., 1500 seconds later), Shp-1 phosphatase was added to the solution. The luminescence decreased due to the dephosphorylation of P1-pY. After 300 seconds, Src kinase was again added (at the same time, sodium orthovanadate Na_3_VO_4_ was added to the reaction mixture to deactivate Shp-1 phosphatase). The luminescence intensity increased again. Apparently, the second Src kinase reaction was successfully monitored, even when the mixture was so complicated and contained many components (the products of the foregoing phosphorylation and dephosphorylation reactions, the deactivated Shp-1, and other remaining reagents).

Most of previous kinetic studies on these enzymatic reactions involved either radiolabeling of the peptide/ATP or chemical labeling of peptide with chromophores. The labeling procedures are time-consuming and still, more importantly, could cause nonnegligible perturbations on the enzymatic reactions. Compared with these methods, the present methods using the Tb^III^ complexes are advantageous in that no labeling is required and kinetic information can be straightforwardly obtained. Simply by adding the Tb^III^ complexes to the reaction mixture and monitoring the photoluminescence, the time-courses of reactions can be directly obtained* in situ* in real-time. Accordingly, detailed kinetic results are precisely obtained, even when the enzymatic systems are highly complicated and the reaction rates do not strictly obey simple Michaelis-Menten equation (e.g., allosteric control in the enzymatic systems and inhibition by other products).

## 5. Quantitative Evaluation of PTK and PTP Inhibitors Using Tb^**III**^-Based Chemical Sensor [69]

There are many kinds of protein tyrosine kinases (PTKs) and protein tyrosine phosphatases (PTPs) in our bodies. Each of them takes an important role in the corresponding reaction and is strongly related to various diseases. Thus, inhibitors on a predetermined enzyme among these PTKs/PTPs have been regarded as promising targets for drug discovery and the subject of growing interest. This section presents the application of binuclear Tb^III^ complexes to screening of inhibitors from a pool of candidates. As described above, the binuclear Tb^III^ complexes can visualize the enzymatic phosphorylation and dephosphorylation in a real-time fashion. Accordingly, the inhibition activity can be evaluated in terms of both kinetic aspects and static aspects, providing new kinds of information to these fields. In this section, the specificity and activity of well-known inhibitors on PTKs and PTPs were determined by using Tb^III^ complexes and compared with the literature data primarily to confirm the validity of method.

### 5.1. Inhibitors on PTK

**Figure 8:**
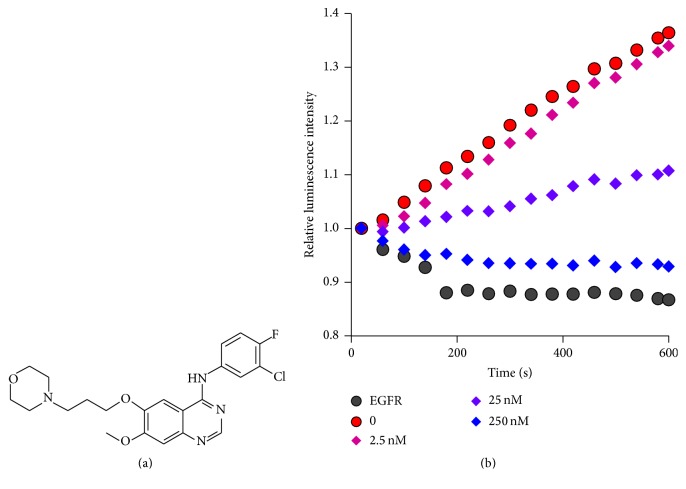
Real-time monitoring by Tb^III^_2_-L^1^ on the gefitinib inhibition of P1 phosphorylation by protein tyrosine kinase EGFR. (a) The structure of the inhibitor gefitinib. In (b), gefitinib was added to the solution containing P1, MnCl_2_, EGFR kinase, and Tb^III^_2_-L^1^, and the reaction was started with addition of ATP. Gray: control without EGFR; red: control without gefitinib. Reproduced with permission from [69]. Copyright 2014, Springer.

Using Tb^III^_2_-L^1^ as a chemical sensor, the inhibitory effects of PTK inhibitors on three kinds of PTKs (Src, Fyn, and EGFR) were analyzed. Among the PTK inhibitors investigated, staurosporine is a general kinase inhibitor with minimum selectivity [131, 132]. On the other hand, gefitinib is effective only in inhibiting EGFR [133], and dasatinib strongly inhibits the other two PTKs [134]. Imatinib is inactive to all of them [135]. To the solution containing P1, MnCl_2_, Tb^III^_2_-L_1_, and a PTK, gefitinib was added, and the reaction was started with the addition of ATP. The inhibitory effects were visually analyzed in terms of the real-time kinetics. Gefitinib drastically suppressed the enzymatic reaction of EGFR (its target PTK) depending on its concentration (Figure 8). When [gefitinib] = 25 nM, the activity of EGFR was reduced to approximately half. In contrast, this inhibitor was much less effective to the other two PTKs (Src and Fyn). Even with [gefitinib] = 25 *μ*M (1000-fold larger than EGFR case), they showed sufficient activity. The inhibition specificity was completely identical with the known specificity.

**Figure 9:**
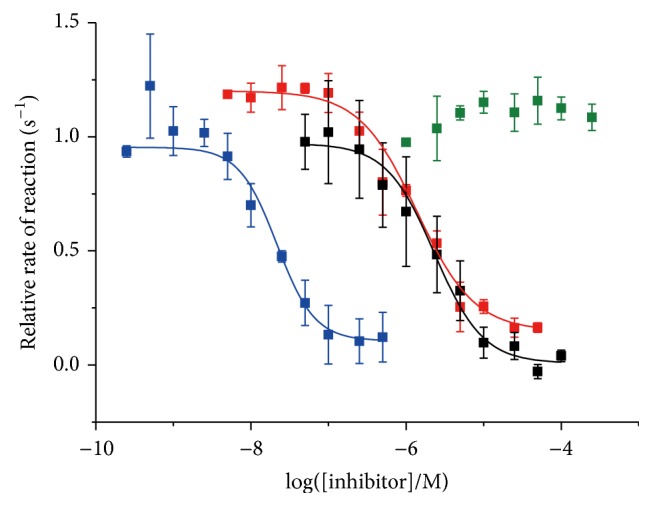
Plots of the rates of EGFR kinase-catalyzed phosphorylation of P1 versus the concentrations of various inhibitors. The rates of phosphorylation of P1 by the kinase were determined in the presence of dasatinib (red), gefitinib (blue), imatinib (green), and staurosporine (black) by the method in Figure 8. The IC_50_ values of the inhibitors, calculated by fitting these sigmoidal curves, are presented in Table 1, together with the values on Src and Fyn kinases. Reproduced with permission from [69]. Copyright 2014, Springer.

Still more quantitative assay of the activity of inhibitors in terms of the half maximal inhibitory concentration (IC_50_) was also successful (Figure 9). By the use of time-resolved luminescence spectroscopy, high signal-to-noise ratios were accomplished for various combinations of PTKs and their inhibitors, and clear sigmoidal dose-response relationships were obtained. From these curves, the IC_50_ values of these inhibitors to the corresponding enzyme were determined (see Table 1). The specificity of all the inhibitors investigated fairly agreed with that reported in the literature [131]. The present method should be very powerful and promising for various applications, especially when a number of substrates, enzymes, and/or inhibitors are analyzed to screen eminent inhibitors from a pool of candidates.

### 5.2. Inhibitors on PTP

The inhibitory effects of Na_3_VO_4_ [136] and *α*-bromo-4′-hydroxyacetophenone [137] on PTPs (Shp-1 and PTP1B) were investigated using Tb^III^_2_-L^1^. The luminescence intensity gradually decreased as the enzymatic dephosphorylation proceeded and the concentration of pTyr decreased. Upon the addition of inhibitors, the magnitude of this decrease became smaller. Consistently, the change in luminescence intensity by PTP-induced dephosphorylation of P1-pY depended on the inhibitor concentration. The IC_50_ values, calculated from the sigmoidal curves, were completely consistent with known selectivity of these inhibitors.

## 6. Attempts to Improve the Detection Sensitivity: Conjugation of Binuclear Tb^**III**^ Complexes to Substrate Peptides [38]

**Figure 10:**
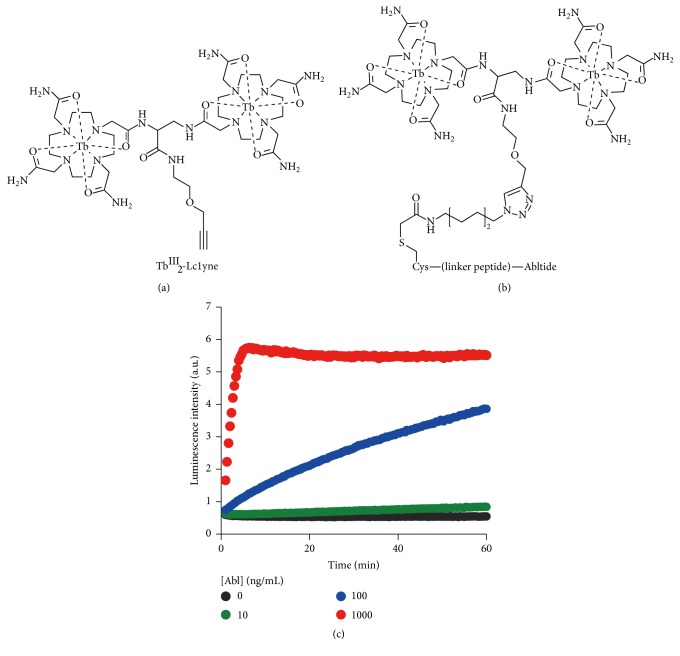
(a) Tb^III^_2_-Lc1yne and (b) its conjugate with Abltide peptide prepared by click reaction. In (c), phosphorylation of Abltide by tyrosine kinases Abl was monitored in real-time using the conjugate by measuring time-resolved luminescence at 545 nm. The concentrations of Abl were 1000 (red), 100 (blue), and 10 ng/mL (green). Conditions: [the conjugate] = 5 *μ*M, [ATP] = 100 *μ*M, [MgCl_2_] = 1 mM, and [NaCl] = 7.5 mM. Reproduced with permission from [38]. Copyright 2015, American Chemical Society.

As described above, both phosphorylation of tyrosine by PTK and dephosphorylation by PTP are visually monitored simply by adding the binuclear Tb^III^ complexes (Tb^III^_2_-L^1^ and Tb^III^_2_-L^2^) to the reaction mixtures. The method is simple, straightforward, and useful. However, the detection sensitivity is in some case rather small, especially when the peptide substrates are positively charged. There, the association of peptides with the positively charged Tb^III^ complexes is suppressed by electrostatic repulsion, and thus the energy-transfer from pTyr to Tb^III^ does not satisfactorily proceed (note that bindings are primarily due to electrostatic interactions). As a general strategy to overcome these drawbacks, substrate peptide and the Tb^III^ complex were covalently connected, and the intermolecular association between them was converted to more efficient intramolecular one. In order to facilitate the synthesis of these conjugates, a new binuclear Tb^III^ complex (Tb^III^_2_-Lc1yne; Figure 10(a)) was prepared by attaching an alkynyl group to Tb^III^_2_-L^1^. Separately, an azido group was bound through a linker to a cysteine residue which was additionally introduced to the peptide substrate. The conjugate was easily obtained by click reaction between the alkyne in Tb^III^_2_-Lc1yne and the azido group in the peptide (Figure 10(b)). This strategy can be applied to various peptide substrates without significant limitation in the structures.

The Tyr phosphorylation of Abltide (Glu-Ala-Ile-Tyr-Ala-Ala-Pro-Phe-Ala-Lys-Lys-Lys; a well-known substrate for Abl kinase) [130] by Abl kinase was monitored (Figure 10(c)). At the pH for the phosphorylation, this peptide bears net charge of +2 and the interaction with Tb^III^_2_-L^1^ was too weak. All the attempts to monitor its phosphorylation using nonconjugated Tb^III^_2_-L^1^ complex were unsuccessful. Accordingly, a cysteine residue was introduced to the N-terminus of Abltide, and an azido was bound thereto. The conjugation of this modified peptide with Tb_2_-Lc1yne by click chemistry proceeded smoothly. When the resultant conjugate was treated with Abl kinase in the presence of ATP, the luminescence intensity increased time-dependently due to the phosphorylation of Tyr. When the peptide concentration was 300 nM, the signal-to-noise ratio was 15.1, being sufficient for detailed quantitative analysis of the reaction. The signal for the Tyr phosphorylation was clearly observed even when the peptide concentration was decreased down to 50 nM. Similar results were obtained when Src was used as PTK and the Tyr phosphorylation of Abltide was successfully monitored. In addition to the N-terminus conjugation, the Tb_2_-Lc1yne can be also conjugated to the C-terminus of Abltide. The advantage of the present conjugation strategy is conclusive.

The effect of the distance between the Tb^III^ complex and the pTyr on the monitoring activity of the conjugate was analyzed by changing the length of linker peptide between the N-terminus of Abltide and the cysteine residue (1 to 5 amino acids). The rate of phosphorylation was almost the same for all the five conjugates. The Tb^III^ complex bound to the Abltide imposes minimal steric hindrance on the enzymatic reaction. Furthermore, the luminescence increased in almost the same magnitude (10-fold) upon the phosphorylation. Apparently, the structure of the complex formed between the Tb_2_-Lc1yne and the pTyr residue in the peptide is similar irrespective of the length of the linker peptide. This ensures the applicability of this strategy to various substrates without strict structural restriction.

## 7. Conclusions

The importance of phosphorylation of tyrosine residues in proteins and their dephosphorylation has been well recognized, and chemical sensors to monitor this phosphorylation selectively have been attracting interests. Recently, significant progress has been made in this field. A DOTAM complex of Tb^III^ showing very high selectivity to pTyr has been developed. The photoemission from Tb^III^-DOTAM complex is notable only when pTyr exists in the solutions. There, the benzene ring of pTyr functions as an antenna and transfers its photoexcitation energy to the Tb^III^ ion as the emission center. Accordingly, the emission is selective to pTyr, since nonphosphorylated tyrosine cannot efficiently bind to the Tb^III^ complex, and neither phosphoserine nor phosphothreonine can satisfactorily provide an antenna effect. Furthermore, the binding of bulky cosolutes (e.g., nucleotides and nucleic acids) to Tb^III^ is suppressed by the steric hindrance of DOTAM. By the use of time-resolved luminescence analysis, only the long life-time luminescence from Tb^III^ is analyzed and high signal-to-noise ratios are accomplished.

Binuclear Tb^III^ complexes (Tb^III^_2_-L^1^ and Tb^III^_2_-L^2^), in which two Tb^III^-DOTAM complexes are connected through the linkers in the ligands, are far more effective in the detection of pTyr than the monomeric Tb^III^ complex. The increase in the sensitivity is primarily ascribed to the stronger binding of pTyr to these complexes, due to enhanced electrostatic interactions between them. With the use of these complexes as sensors, phosphorylation of tyrosine by protein tyrosine kinases and dephosphorylation by protein tyrosine phosphatases are visualized* in situ* in a real-time fashion. Furthermore, the activities of various inhibitors on these enzymes are quantitatively evaluated by the Tb^III^ complexes. This method should be useful in screening highly eminent inhibitors from a number of candidates. These enzymes take crucially important biological roles so that the information obtained by these studies should lead to development of new drugs for the therapy of relevant diseases. By immobilizing these Tb^III^ complexes to some solid supports, the applications of the present methods should be further facilitated and widened.

## Supplementary Material

By using binuclear Tb^III^ complexes, phosphorylation of tyrosine in peptides by protein tyrosine kinases (PTKs) and dephosphorylation by protein tyrosine phosphatases (PTPs) can be successfully visualized in a real-time fashion. The activities of various inhibitors on these enzymes are quantitatively evaluated, indicating a strong potential of the method to efficient screening of eminent inhibitors from a number of candidates.

## Figures and Tables

**Table 1:** IC_50_ of PTK inhibitors.

IC_50_ (nM)	Dasatinib	Gefitinib	Imatinib	Staurosporine
Src	12 ± 0.96	>5000^a^	—^b^	310 ± 28
Fyn	26 ± 2.2	>2000^a^	>10000^a^	260 ± 19
EGFR	1400 ± 110	22 ± 2.4	—^b^	2300 ± 820

^a^Not determined due to poor curve fitting of weak inhibitors. ^b^Inhibition was not observed.
