# Supplementary material for: Selective Sensing of Tyrosine Phosphorylation in Peptides Using Terbium(III) Complexes
Source: Int J Anal Chem. 2016 Jun 8;2016:3216523. doi: 10.1155/2016/3216523 (PMC4916314; doi:10.1155/2016/3216523)
Supplement: Supplementary file 1 — By using binuclear TbIII complexes, phosphorylation of tyrosine in peptides by protein tyrosine kinases (PTKs) and dephosphorylation by protein tyrosine phosphatases (PTPs) can be successfully visualized in a real-time fashion. The activities of various inhibitors on these enzymes are quantitatively evaluated, indicating a strong potential of the method to efficient screening of eminent inhibitors from a number of candidates. [file 3216523.f1.docx]

By using binuclear Tb^III^ complexes, phosphorylation of tyrosine in peptides by protein tyrosine kinases (PTKs) and dephosphorylation by protein tyrosine phosphatases (PTPs) can be successfully visualized in a real-time fashion. The activities of various inhibitors on these enzymes are quantitatively evaluated, indicating a strong potential of the method to efficient screening of eminent inhibitors from a number of candidates.
